# Switching to a Coronavirus Disease 2019 (COVID-19) Center: Lessons From Ariana Hospital Experience

**DOI:** 10.1017/dmp.2021.128

**Published:** 2021-04-30

**Authors:** Nidhal Belloumi, Imene Bachouch, Fatma Chermiti Ben Abdallah, Soraya Fenniche

**Affiliations:** 1 Pulmonology Department “Pavilion 4”, Abderrahman Mami Hospital, Ariana, Tunisia; 2 Faculty of Medicine of Tunis, University of Tunis El Manar, Tunis, Tunisia

**Keywords:** COVID-19, crisis units, communication, health-care worker

## Abstract

After the translating of the worldwide pandemic coronavirus disease 2019 (COVID-19) disease from South East Asia to Europe, North African countries accelerate their steps to follow WHO guidelines to prepare the outbreak response. In March 2020, the Tunisian Ministry of Health switched Abderrahmen Mami Hospital to a COVID-19 center. The main objectives were management of patients but also setting-up new rules to permit enough safety for the staff members and harmony between medical, nonmedical, and administrative departments within the facility. Organization and communication during the fast-paced preparation process were crucial to get enough qualified human resources, material resources, and clear procedural texts in place before cases arrived in huge numbers. A group of medical and administrative experts within a central crisis unit brought this challenge into reality.

After the spread of the worldwide pandemic of severe acute respiratory syndrome coronavirus 2 (SARS-CoV-2) and coronavirus disease 2019 (COVID-19) disease from South East Asia to Europe, North African countries accelerated their steps to follow World Health Organization (WHO) guidelines to prepare the outbreak response. Goals to achieve at a national scale were: identification, tracking, and isolation of imported cases entering Tunisian territory; prevention or reduction of the occurrence of contagion among Tunisian citizens; and preparing health-care centers to stage 3 epidemic state.

On March 2, 2020, the first imported COVID-19 case in Tunisia was declared. On March 23, 2020, Abderrahmen Mami Hospital, in the city of Ariana, was dedicated to the management of COVID-19 patients.^1^ The emergency department in Mahmoud El Matri Hospital was designated to ensure the targeted screening for COVID-19 in suspected patients, severity assessment, and the guidance of patients toward other departments if necessary.

## Methods

The hospital previously contained pulmonology, cardiology, chest surgery, and intensive care departments. Guidance rules were defined and written to fulfill its new designation as a COVID-19 center. During epidemics, a coordinated and multidisciplinary management between specialists in infectious diseases, intensive care units (ICUs), and infection prevention and control, but also the hospital management, is of paramount importance to provide optimal care.

In this manuscript, we describe in detail all preparative measures and the new organizational setup in this medical center to enable the switch to an uncommon health-care regimen. This study provides a rich description of phenomena and enhances the understanding of the context of events as well as the events themselves during the first COVID-19 wave.

To report faithfully all the previous, authors examined all procedural texts and notes provided by the medical council, all administrative assignments recorded as ordinary, exceptional, or temporary during the first quarter of 2020.

### Set Up of a Steering Crisis Unit and Derivative Sub-units With Clear Missions

In the beginning, an important concern voiced by staff was a lack of preparedness for COVID-19. Once the National Body for Health Assessment and Accreditation posted its main manual for COVID-19 management,^2^ steering crisis unit members had to provide their professional expertise and up-to-date all-written guidance on procedures for management of suspected and confirmed cases of COVID-19 within the hospital, considered as a third-line teaching center.

Clinical pathways for screening of incoming symptomatic patients in the hospital entrance were established by a reflection sub-unit. Experts ensure in their plan an avoidance of intersections with COVID-19 free people, a reduction of contagion risk and a safety distance from suspicious cases always respected. Clinical examination areas, waiting spaces, and protective measurements and equipment were precisely described according to WHO guidelines.^3,4^

Daily meetings of national and regional head managers were held each morning to learn about the epidemiological situation in the country, isolated patients in airports, or advanced medical posts near borders, to organize patients dispatching toward specialized centers, to organize donations, and to include more volunteers into existing operational teams. Scientific sub-units regularly discussed problems encountered by doctors, proposed drafting or updating consensus, and tracked feedback from doctors.

An administrative and legal arm was created to watch and review achievements and predictions. Then, real and predicted shortage of supplies were treated efficiently. Donation enhancing, counting and resource distribution is ensured by a specific sub-unit directly connected to the communication and announcement representative members to highlight the real actual needs of the hospital. This inventory management cell managed any shortage in personal protective equipments, chloroquine, or other. Pharmacists could involve the purchasing department easily if necessary.

Training-qualification units transmitted updates regularly (reminding of theorical knowledge, description of the occupational risk, transmission of skills, description or immersion into the new working pathways, application of the distance keeping concept) and surveyed real progress and handling of the procedures among health-care workers (HCWs).

Exchange between all sub-units is made easier through some common members. Prediction was essential to avoid mistakes in the decision-making process.^5^ Predictions were gathered from the previous experience of H1N1 outbreak in 2009, from previous knowledge about severe acute respiratory infections, and from reviewing China and Europe experience against novel coronavirus (nCOV) spread in January and February.

### Protecting HCWs

The fact that HCWs are at risk of infection in the epidemic chain is a critical issue and a real burden in many countries.^6^ Therefore, all possible actions must be taken to control the spread of the infection among HCWs, first by identifying the risk factors for infection and then by taking appropriate measures to reduce these risks.

It was well established that transmission of any viral disease among HCWs is associated with overcrowding, absence of isolation room facilities, environmental contamination, and inadequate awareness of infection prevention practices among both patients and HCWs.^6^ Knowledge of a disease can influence HCWs’ attitudes and practices. Incorrect attitudes and practices directly increase the risk of any infection.^7^ Therefore, COVID-19 training sub-units managed to select well-trained members in every department who facilitated the transmission of new guidelines and rules concerning work in a contaminated isolation area. These sub-units, also, transmitted feedback to the central crisis unit concerning work shift length in every department, team set-up. and sharing responsibilities within all members of the staff. The crisis unit considers these recommendations as much as possible.

From the perspective of scientific prevention and control, HCWs should place a high value on correct removal of the personal protective equipment.^4^ When removing contaminated equipment, such as gowns, gloves, medical masks, and eye protection worn in contaminated or high-risk environments, it is necessary to prevent self- or surface contamination.

Understanding HCWs’ knowledge, attitudes and practices toward possible risk factors helps to predict “at risk staff members” or “higher risk situations.”

There was considerable variation in the use of barrier precautions and personal protective equipment. There was also wide variation in perceptions of security with use of personal protective equipments. HCWs usually avoided receiving various templates, models, or sizes of protective equipment. The key message in these situations was to focus on showing effectiveness of every outfit to wear during a specific medical procedure.

However, inadequate knowledge is not the only risk factor for care. A previous study showed that the causes of higher risk of infection are related to HCW daily practice, the duration and the frequency of their occupational exposure. We defended the concept of front-line personnel while setting clinical and in-patient pathways. They were more exposed during patient intake, screening, inspection, testing, transport, treatment, nursing, specimen collection, or pathogen detection. Therefore, to minimize the contact time with the patient, we applied distance surveillance through a closed-circuit television (CCTV) network linking the infected zone to the staff zone. Nurses considered it a good and safe way to monitor a patient and suggested also to make regular phone calls to patients to minimize the psychological impact of the isolation rules.^8,9^ Greater risk of contagion was observed among housekeeping staff, perhaps related to the complexity of the procedure for transporting patient’s waste and laundry.

Before the outbreak, the pace of work at the hospital was as follows: 1 heterogeneous team of seniors and trainees ensures the morning session (6 h) and a second team ensures the afternoon session (6 h). The evening session (12 h) is provided by 3 teams who pass the evenings in turn at a rhythm of 1 d of 3.

Shifts during the outbreak was as follows: medical staff finish 1 wk of field work on a rhythm of 12-h shifts, followed by 1 wk of confining. Two reverse transcriptase-polymerase chain reaction (RT-PCR) nasopharyngeal staining are done 2 d apart during confining period, before they could go back home for 1 wk of rest.

During the confining period, HCWs were in periodically decontaminated individual hotel rooms. These dedicated hotels had no other guests besides the HCWs. Lockdown measures were set: special agents and bus for transport, daily provided face masks, presence check, and phone-call check. Meal delivery, laundry, and rubbish collecting were directed and done by Ministry of Health teams. During Ramadan (fasting month for Muslims), work shifts were slightly modified to fit the time of fast-breaking, to avoid overwork or nonconscious errors. Meals and water delivery was easier, twice a day.

### Digitalization

Trying to fulfill a safe “paper free” medical activity, there was a real need for more and better documentation as well as digitalization of health-care systems in a short period of time. That entailed shifts in requested competencies and roles of HCWs. During the COVID-19 outbreak, as a result of this data-centric digitalized daily practice and the risk of including medical scribes, this supplementary workload was taken in equal parts by doctors and nurses.

The boundaries between professions as well as their skills, expertise, and task repertoire continuously change in health care due to multiple reasons. In times of shortage of staff, physicians and nurses may hand over routine tasks to other occupational groups, whereas in times of surplus or “imperative need,” they may try to incorporate additional tasks and specialties into their work domain.^10^

During COVID-19 outbreak, trainees and nurses extended and restored their skills in digital compilation. They were able to enter information into the electronic medical record (EMR) or chart at the direction of a physician or practitioner. In practice, a nurse does not and might not act independently but could document the previously determined physician’s or practitioner’s dictation and/or activities. Skilled nurses also assisted the practitioners in navigating through or entering information into the EMR and locating information, such as test results and lab results. They could support work flow and supervise documentation, coding process, and storage of medical or administrative records. The ultimate goal was to allow the physician or practitioner to spend more time with the patient, to spend less time doing clerical work, and to avoid physical displacement from a room to another excessively.

Computed requests for laboratory analyses were activated for the first time during the COVID-19 outbreak. Blood samples were identified by serial numbers or codes corresponding to specific requests made previously by the physician. X-ray explorations requests were computed as well. Less human interaction responding the incoming requests meant avoiding crowds and facilitated maintenance of the sterilization of the closed areas.

### Psychological Support

Citizens’ reaction were classically described after H1N1 spread and large scale experiences, such as tsunami and hurricanes. In addition, according to earlier Chinese and European publications, HCWs reported feeling unsafe while working during the actual outbreak, and expressed uncertainty about the effectiveness of personal protective equipment.^11,12^ So, dealing with the psychological impact of the outbreak on isolated or hospitalized patients, HCWs, and large populations was an important issue to predict.^13–16^

Using a free telephone line, a group of psychologists and psychiatrists ensured that HCWs in our hospital were listened to and supported during or after finishing their anti COVID-19 “duty.” Several cases of infected nurses had to be admitted in their same departments. After discharge, they needed supportive psychotherapy and special care from their doctors.

Psychological support was offered to patients on request, by a group of psychologists using another special free telephone line. Physicians and medical trainees made phone calls to their patients, 2 wk after discharge to follow-up and answer questions if they had any.

### Post Epidemic Strategy

On May 10, 2020, Tunisia scored 0 new case of COVID-19 infection. Twenty days after that, epidemiologists declared total control of the epidemic status: No more new cases, screening of the remaining patients already done, and borders still closed for casual traveling and tourism.

Emergency department then all other departments of cardiology, pulmonology, and imaging returned progressively back to normal activity with strict distancing procedures.

Online platforms or Facebook pages were set to provide appointments to patients. All departments came back to work with 70% of full capacity. Patients came on schedule in small groups every 1 h all over the morning. Waiting areas were re-arranged with fewer chairs. Priority was given to persons with chronic diseases who missed their regular screening appointments. Experts within the reflection sub-unit evaluated the whole experience and wrote procedures predicting a second wave of COVID-19 infection in Tunisia

## Discussion

Ariana hospital was the only third-line medical center entirely dedicated to COVID-19 management in Tunisia. The other hospitals in Tunisia followed the classic plan used worldwide.^6,17,18^ They developed triage at their entry gates leading to COVID-19 or COVID-free circuits with no intersections. They built, also, isolated waiting zones and small COVID-19 admission pavilions. Ariana hospital, instead, included the largest intensive care department entirely dedicated to severe cases of COVID-19 patients in Tunisia. There was also 7 pulmonology departments for mild cases, a cardiology department containing an angioplasty unit, an imaging department with 3 access plan to facilitate decontamination after every exploration, and a surgery department dealing with all types of surgical or gynecological emergencies in COVID-19 patients. In Ariana hospital, there was no need to set up a COVID-19 versus COVID-free physical separation. Triage is performed in another hospital nearby, allowing medical units to receive infected patients already diagnosed, with severity assessment quite done. Nevertheless, the transport of the patients from a center to another was found to be a critical point to anticipate. Patient movement circuits to avoid infectious spread had to be organized by the infection prevention and control (IPC) team and forwarded to involved HCWs. The inventory department had to adapt to a rapidly growing demand and provide the personnel with the necessary equipment for treatment.

Infection among personnel of the hospital during the first COVID-19 wave was sporadic. We registered 4 cases with mild symptoms and 4 asymptomatic cases of COVID-19 infection. Taking into consideration the facility size (1400 employees in a third-line teaching hospital), infection rate would be near zero.

On the other hand, international data and experience exchange was entrusted to a group of seniors. This quality control sub-unit made daily debriefing sessions in small groups or by means of web-conferences to make sure of the unfolding of all processes: pretriage, targeted screening, nasopharyngeal swab performing, buffering and waiting zone management, and exchanging between various implicated departments in and out of the hospital.^19^

Centralization of management decisions was an administrative choice coming from the Ministry of Health. The national crisis unit was connecting to sectorial units, which are themselves bringing data from hospital crisis units. Resource managing and consumption were directly under their control to avoid any standstill and to predict shortage. The public sector had a full priority to get supplies of reactant products for chemical testing, hydroxychloroquine pills for oral route therapy, and individual protective equipments. This schema had proven efficient in previous similar experiences.^20–22^

Such experiences have succeeded in ensuring proper management of all directed patients who need to be hospitalized. The hospital had reached its maximum capacity of ICU reserved places, but never reached its limits to receive mild cases.

Worldwide, we found no great difference between high- or low-income countries strategies during outbreak onset. After closing the borders, it all depends on rapidity to set up all measurements and the real size of the pandemic state in a national scale. A major work has been conducted to improve the resilience of infrastructure in terms of building new facilities, ensuring supplies while decision-makers were trying to limit the monetary expenditure. Main strategic decisions were congruent to abilities of a middle-income country.^17,20,23^

In Tunisia, the maximum number was 58 per day in April 4, 2020, and then dropped quickly. The situation was always described as under control. During the first wave of COVID-19 spread, the so-called big Tunis governorates (approximately 25% of the entire population) has recorded a mortality rate of 0.66% within 150 officially recorded cases. Sfax is the city with the second highest population density hosting almost 9% of the entire population in the country. In this city, 14 COVID-19 cases were officially registered on March 29 with 3 lethal outcomes.

However, the tests have been performed only for persons with severe disease symptoms and after evident contact to infected patients. Indeed, the maximum recorded tests made in Tunisia are 724 daily. The official statistics revealed that only 10% of tested people were COVID-19 positive. Taking this number in consideration, more than 45,000 tests have to be performed to limit the virus propagation before stopping containment. On May 10, Tunisia had zero new infections registered. The total number of patients officially declared was 1032. Active contagious cases were 327 patients. Management of the first wave of the outbreak was based on predictions from the European experience.^24–26^ The central directorate of health got scenarios of the next 2-3 wk and tried to apply measurement strategies for a stage 3 pandemic state, whereas Tunisia was still in stage 2. Getting 1 step forward permitted Tunisia to stop the first wave of coronavirus spread. Therefore, assuming that the real number of infected persons is 4 to 10 times higher, authors and decision-makers waited wisely at least to get beyond 150,000 tests performed before deciding on stopping containment. The Ministry of Health focused then to plan perfect modalities of re-opening borders, re-activate safely the touristic activity, manufacturing, and normal administrative activities.

## Conclusions

All health structures in Tunisia were prepared to face an increasing number of patients with COVID-19 and had to anticipate the consequences, including the need for more beds, more ICUs, ventilators, and trained HCWs. To face the outbreak, hospitals have to anticipate the consequences of COVID-19 on all the departments, including indirect impact on noninfected patients. Ariana Hospital switched entirely to a COVID-19 dedicated center and prepared for a fight against COVID-19 that would last for months. A full commitment of health authorities and hospital management staff led to an organized communication with the wider public.

In developing low-income countries, a better response to COVID-19 outbreak was noticed in countries that already made progress in several socio-economic fields: digitalization, continuous recycling process, popular awareness, welfare recipient assistance, and enhancing the civil society role. The balance between material resources preservation, security of field effectors, and providing of medical care was the subject of daily discussion by health system experts.

Managing a crisis throughout the hospital generally relies on the existence of an updated “crisis plan,” a dynamic revision process led by doctors and a skilled IPC team. This makes it possible to translate and personalize, in the field, ministerial decisions efficiently.
